# Functionality of C-Reactive Protein for Atheroprotection

**DOI:** 10.3389/fimmu.2019.01655

**Published:** 2019-07-16

**Authors:** Sanjay K. Singh, Alok Agrawal

**Affiliations:** Department of Biomedical Sciences, James H. Quillen College of Medicine, East Tennessee State University, Johnson City, TN, United States

**Keywords:** C-reactive protein, low-density lipoprotein, cholesterol, atherosclerosis, amyloid

## Abstract

C-reactive protein (CRP) is a pentameric molecule made up of identical monomers. CRP can be seen in three different forms: native pentameric CRP (native CRP), non-native pentameric CRP (non-native CRP), and monomeric CRP (mCRP). Both native and non-native CRP execute ligand-recognition functions for host defense. The fate of any pentameric CRP after binding to a ligand is dissociation into ligand-bound mCRP. If ligand-bound mCRP is proinflammatory, like free mCRP has been shown to be *in vitro*, then mCRP along with the bound ligand must be cleared from the site of inflammation. Once pentameric CRP is bound to atherogenic low-density lipoprotein (LDL), it reduces both formation of foam cells and proinflammatory effects of atherogenic LDL. A CRP mutant, that is non-native CRP, which readily binds to atherogenic LDL, has been found to be atheroprotective in a murine model of atherosclerosis. Thus, unlike statins, a drug that can lower only cholesterol levels but not CRP levels should be developed. Since non-native CRP has been shown to bind to all kinds of malformed proteins in general, it is possible that non-native CRP would be protective against all inflammatory states in which host proteins become pathogenic. If it is proven through experimentation employing transgenic mice that non-native CRP is beneficial for the host, then using a small-molecule compound to target CRP with the goal of changing the conformation of endogenous native CRP would be preferred over using recombinant non-native CRP as a biologic to treat diseases caused by pathogenic proteins such as oxidized LDL.

## Introduction

C-reactive protein (CRP) is a pentamer of identical subunits which functions in two different structural states, as native pentameric CRP (native CRP) in normal physiological environment and as non-native pentameric CRP (non-native CRP) in localized pathological and inflammatory environments (1–7). During making of CRP in the liver, first, the five subunits fold to almost a native core and the single C-terminal helix is correctly positioned. Then, the intrachain disulfide bond between Cys^36^ and Cys^97^ is formed. Further folding of the subunit is driven by the newly formed disulfide bond and Ca^2+^-binding. Finally, CRP is assembled as pentamers and secreted into the circulation (8). It has been shown that recombinant CRP is not assembled and not secreted from the transfected cells if there is a mutation in the region coding for its Ca^2+^-binding site (9). When CRP enters an inflammatory microenvironment and is exposed to pathological conditions, the data obtained from *in vitro* experiments suggest that the pentameric structure of CRP is converted from its native conformation to a non-native conformation (2, 10, 11). Whether it is native CRP or non-native CRP, binding of CRP to a ligand causes dissociation of pentameric CRP and generation of monomeric CRP (mCRP) on the surface of the ligand (10).

Atherosclerosis is an inflammatory disease caused by the deposition and subsequent modification of low-density lipoprotein (LDL) in artery walls (12–14). Modified LDL is atherogenic: it is recognized and engulfed by macrophages to form LDL-loaded foam cells that contribute to the development of atherosclerotic lesions (14–16). It has been suggested that in areas in which inflammation takes place, including in atherosclerosis, the pH may be acidic due to hypoxia, lactate generation, activated macrophages and proton generation (17–21). Since CRP has been found to localize with LDL and macrophages in atherosclerotic lesions in both humans and experimental animals, CRP has been implicated in modulating the pathogenesis of atherosclerosis (22–26). Here, we review the literature on the structure-function relationships of CRP *in vitro* and *in vivo* as applied to atherosclerosis and conclude that CRP plays a defensive role in the pathogenesis of atherosclerosis (27, 28).

## Functions of CRP (native CRP) in Atherosclerosis

CRP, in its native pentameric conformation and in the presence of Ca^2+^, binds to cells and molecules with uncovered phosphocholine (PCh) groups, such as the membrane of damaged cells and platelet-activating factor (29–31). Each subunit in the pentamer has a PCh-binding site. The three-dimensional structure and mutagenesis of the PCh-binding site revealed that Glu^81^, Phe^66^ and Thr^76^ are critical for creating the pocket on CRP to bind and accommodate PCh (32–35). Once CRP is bound to a PCh-containing ligand, it activates the classical complement pathway (36).

Many kinds of modifications can occur to deposited LDL in arteries; however, two types of modified LDL prepared *in vitro*, oxidized LDL (ox-LDL) and enzymatically-modified LDL (E-LDL), are mostly used in experiments to define the role of CRP in atherosclerosis (37–39). Since the PCh groups present in LDL are exposed in E-LDL, CRP is able to bind to E-LDL in a Ca^2+^-dependent manner (40, 41). CRP does not bind to ox-LDL; however, CRP can bind to ox-LDL if LDL is oxidized enough to expose its PCh moiety (42–45). If CRP binds to ox-LDL independent of the exposure of PCh on ox-LDL, it would be possible only in a pathological milieu that can affect CRP structurally (10, 11). CRP has also been shown to bind to complexes consisting of ox-LDL and β2-glycoprotein I (46, 47). CRP also binds to cholesterol crystals and it has been shown that CRP is located mainly in the cholesterol-rich necrotic core in atherosclerotic lesions (48). It has been shown that CRP also binds to LOX-1 which is a receptor for ox-LDL (49, 50).

CRP, ox-LDL and E-LDL all are known to be involved in interrelated pathophysiological pathways including in the formation of LDL-loaded macrophage foam cells (16, 51). However, the literature on the effects of CRP on the formation of foam cells has been controversial. Since CRP was found to be located intracellularly in foam cells, it was hypothesized that CRP complexes with LDL, enhances the binding of LDL to macrophages, and thus facilitates the cellular uptake of LDL along with CRP (52–57). When pure complexes of CRP and E-LDL were used for treatment of macrophages, it was found that CRP-bound E-LDL was unable to form foam cells, clearly suggesting for the first time that CRP possesses the ability to prevent the formation of foam cells (58). Indeed, in another study, the complexes of CRP and LDL were found to be unable to enter macrophages (59). In addition, when endothelial cells and a third type of modified LDL, acetylated LDL, were used in foam cell experiments, mCRP was found to decrease the uptake of acetylated LDL by endothelial cells (60). In another study employing endothelial cells as a model for foam cell formation, CRP was found to increase LDL transcytosis across endothelial cells (61). mCRP has also been shown to decrease uptake of ox-LDL by macrophages and it has been proposed that the interaction of mCRP with ox-LDL may contribute to retardation of the foam cell formation by reducing the aggressive macrophage response to ox-LDL (43, 62). Additionally, it has been proposed that mCRP may exert a protective role by facilitating the clearance of retained native LDL from extracellular space, and thus lower the risk of LDL modifications (43). But, since foam cell formation is inhibited whenever CRP is complexed with modified LDL such as CRP-E-LDL and mCRP-acetylated LDL, it has been proposed that if each LDL molecule retained in the arterial wall becomes CRP-bound, the development of atherosclerosis should be retarded (58).

Besides the effects of CRP on the formation of foam cells, other consequences of the interactions between CRP and modified LDL have been reported, although it is unclear whether it was ensured that CRP was free of spontaneously generated mCRP. CRP, after binding to LDL, causes charge modification of LDL (59). The production of proinflammatory cytokines by macrophages decreases when the cells are treated with a combination of CRP and ox-LDL (62). CRP inhibits the susceptibility of copper-induced oxidation of LDL, that is, once CRP is bound to ox-LDL, further oxidation is prevented, and CRP does so by prolonging the time it takes for copper ions to oxidize LDL (63, 64). By sequestering minimally modified LDL (mmLDL), CRP can prevent binding of mmLDL to monocytes and attenuate mmLDL-induced monocyte adhesion and activation (65). CRP was also found to suppress the proatherogenic effects of macrophages when bound to lysophosphatidylcholine present in ox-LDL and inhibit the association of ox-LDL to macrophages; this effect may in part retard the progression of atherosclerosis (66). These findings suggest that not only does CRP prevent foam cell formation but also reduce the proinflammatory effects of modified LDL and foam cells.

Human CRP, mouse CRP and rabbit CRP have all been used to determine the effects of CRP on the development of atherosclerosis. For human CRP, three different murine models of atherosclerosis, *ApoE*^−/−^ mice, *LDLr*^−/−^ mice and *ApoB*^100/100^*LDLr*^−/−^ mice, and a rabbit model of atherosclerosis have been employed. CRP was either transgenic or passively administered. In most studies employing *ApoE*^−/−^ mice, CRP was found to be neither proatherogenic nor atheroprotective: both passively administered human CRP and transgenically expressed human CRP had no effect on the development, progression, or severity of atherosclerosis (67–71). In two studies employing *ApoE*^−/−^ mice, CRP slightly worsened the disease (72, 73). In another study employing *ApoE*^−/−^ mice, CRP promoted early changes of atherosclerosis by directly increasing the transcytosis of LDL across endothelial cells and increasing LDL retention in vascular walls (61). In *LDLr*^−/−^ mice also, there was no effect of CRP on the development of atherosclerosis (74). When *ApoB*^100/100^*LDLr*^−/−^ mice were employed, which are rich in LDL and develop human-like hypercholesterolemia, CRP slowed the development of atherosclerosis, suggesting an atheroprotective role of CRP (75). In the rabbit model of atherosclerosis also, there was no effect of transgenic human CRP on either aortic or coronary atherosclerotic lesion formation (76). CRP-deficient mice were employed to observe any possible role of endogenous murine CRP in atherosclerosis (77). In both *ApoE*^−/−^*CRP*^−/−^ and *LDLr*^−/−^*CRP*^−/−^ mice, the size of atherosclerotic lesions was either equivalent or increased when compared to that of *ApoE*^−/−^ and *LDLr*^−/−^ mice, suggesting that murine CRP had the ability to mediate atheroprotective effects (77). Besides human and murine CRP, the effect of rabbit CRP on the development of atherosclerosis in rabbits has also been investigated by using CRP antisense oligonucleotides (78). CRP antisense oligonucleotide treatment led to a significant reduction of CRP levels in rabbits; however, both aortic and coronary atherosclerotic lesions were not significantly changed, suggesting that inhibition of plasma CRP does not affect the development of atherosclerosis in rabbits (78). The combined data suggest that native CRP was either incapable or only partly capable for protecting against atherosclerosis in animal models.

## Functions of Non-native Pentameric CRP (Non-native CRP) in Atherosclerosis

In the presence of a biological protein modifier, the structure of CRP is altered leading to the production of non-native CRP which ultimately generates mCRP (1–5, 79). Dissociation of CRP to mCRP thus involves an intermediate stage of non-native CRP, and it has been shown that antibodies specific for mCRP react with non-native CRP also (1). There are several modifiers of CRP structure. CRP is modified in the presence of abundant damaged cell membranes (1). The binding of CRP to activated platelets and apoptotic cells has also been shown to change the structure of CRP to generate mCRP (80, 81). CRP, by binding to cell-derived microvesicles, undergoes a structural change without disrupting the pentameric symmetry and constitutes the major CRP species deposited in inflamed tissue (4). mCRP has also been seen deposited at burn wounds having necrotic and inflamed tissue (82). Acidic pH condition modifies CRP (10, 83). CRP is also modified by hydrogen peroxide and hypochlorous acid (11, 84). Hypochlorous acid modifies CRP by oxidation and chlorination of amino acids, leading to protein unfolding, greater surface hydrophobicity and the formation of aggregates (84). These findings suggest that when CRP enters an inflammatory microenvironment and is exposed to pathological conditions, the structure of CRP is changed first to a non-native pentameric conformation leading to complete dissociation of CRP and generation of mCRP.

Except for binding to PCh, the recognition functions of non-native CRP are different from those of CRP (2, 7). One function of CRP in its non-native pentameric conformation is to bind to modified LDL irrespective of the presence of PCh and Ca^2+^. Unlike CRP, non-native CRP readily binds to ox-LDL regardless of the extent and nature of the oxidation status (10, 11). To E-LDL, non-native CRP binds more avidly than CRP does (83). It has also been shown that, in the absence of Ca^2+^, a new lysophosphatidylcholine-binding site located on the opposite side of the known PCh-binding site becomes functional (85, 86). The binding to and actions of CRP on endothelial cells also requires a conformational rearrangement in CRP (87). Taken together, the deposition of CRP and its co-localization with LDL in atherosclerotic lesions indicate the presence of non-native CRP at the lesions. Besides PCh, the other moieties on LDL molecules that interact with CRP include apolipoprotein B and cholesterol. However, the moiety on modified LDL with which non-native CRP interacts is unknown (88–90). The binding site on non-native CRP for modified LDL has not been elucidated as yet either. It has been proposed though that the binding site may involve amino acid residues participating in the formation of intersubunit contact region since this region is buried in CRP and accessible in non-native CRP (2, 10). In addition, a single sequence motif called the cholesterol binding sequence, from amino acid residue 35 to 47, has been found to be responsible for mediating the interactions of mCRP with diverse ligands. The versatility of the cholesterol binding sequence appears to originate from its intrinsically disordered conformation (91).

Although the investigations to determine the effects of CRP on the development of atherosclerosis in animals provide conflicting results, a study employing mCRP in *ApoE*^−/−^ mice indicated that mCRP was atheroprotective (73). Additionally, the data obtained from *in vitro* experiments raised hopes that non-native CRP may be more atheroprotective than CRP, considering the difference between the LDL-binding recognition functions of CRP and non-native CRP. Employing site-directed mutagenesis, it was possible to create CRP mutants capable of binding to ox-LDL without the requirement of any further structural change, and one such mutant has been reported earlier (92). Recently, the impact of such a CRP mutant on the development of atherosclerosis was evaluated employing the *LDLr*^−/−^ mouse model of atherosclerosis (93). The development of atherosclerotic lesions in the whole aorta was reduced in mice receiving mutant CRP that had a non-native pentameric structure. Considering the findings made on all forms of CRP structure, it seems clear that CRP is an atheroprotective molecule (93).

## Proinflammatory Functions of Ligand-Bound mCRP

Once CRP, either native or non-native, is bound to certain types of ligands, mCRP is generated on the surface of the ligand, due to complete dissociation of the five subunits of CRP. It has been shown that the binding of non-native CRP to immobilized protein ligands results in expression of mCRP epitopes and that mCRP cannot be detached from the ligand (10). Thus, mCRP is not a free molecule; instead, mCRP is always ligand-bound and found in CRP-derived debris. The presence of mCRP can be detected at the sites where CRP-ligands are present. The detection of autoantibodies against mCRP provided further evidence for the *in vivo* existence of non-native CRP and mCRP, probably ligand-bound (94–96). The mCRP form is the predominant form of CRP existing in atherosclerotic lesions (80, 97–100). It has also been shown that the expression of proinflammatory properties of CRP requires sequential conformational changes beginning with the loss of pentameric symmetry, followed by reduction of the intrasubunit disulfide bond, generating mCRP (87, 101). Since free mCRP is proinflammatory in *in vitro* experiments, it can only be assumed that ligand-bound mCRP may also be proinflammatory. Ligand-associated mCRP must be removed along with the ligand.

## CRP, Statins, and Atherosclerosis

Statins, the inhibitors of a key enzyme in the cholesterol biosynthesis pathway, are used in humans as cholesterol-lowering drugs (102). However, statins also lower CRP levels in humans and human CRP-transgenic mice (103–108). Statins lower CRP levels independently of their cholesterol-lowering activity (103, 104). Statins lower CRP by inhibiting the biosynthesis of CRP by hepatocytes (109, 110). Nitric oxide also inhibits the biosynthesis of CRP (109). It is possible that nitric oxide acts as the mediator of the CRP-lowering effect of statins, since statins are known to generate nitric oxide production (109–112). Because CRP is beneficial, to get rid of CRP from the circulation is not a good idea; a drug that can lower cholesterol levels, but not the CRP levels, should be of choice over statins which lower both (113, 114).

## Conclusions

CRP appears in the body in response to inflammation and CRP requires exposure to an inflammatory milieu to change its structure and execute functions (2, 115). We have hypothesized earlier that one of the functions of CRP at sites of inflammation is to sense the inflammatory microenvironment, change its own structure in response but remain pentameric, and then bind to pathogenic proteins deposited at those sites (11). CRP does not show an effect on the development of atherosclerosis likely because the inflammatory microenvironment in the arterial wall in animal models of atherosclerosis may not be appropriate in terms of pH and redox conditions and, therefore, the structure of CRP remains unchanged. Consistent with this hypothesis, a CRP molecule which was modified *in vitro* and was capable of binding to atherogenic LDL, did reduce the development of atherosclerosis in mice (93). Thus, CRP has atheroprotective functions displayed by its non-native pentameric form. It has also been proposed that CRP-mediated lipoprotein removal likely underlies the regression of early lesions and perhaps CRP should be considered as an antiatherogenic agent (39).

Non-native CRP binds not only to atherogenic LDL but to all immobilized proteins, including proteins that might be deposited in the host body or recruited on pathogenic surfaces (10, 116). We have suggested previously that deposited, aggregated and conformationally denatured proteins expose a CRP-ligand, regardless of the protein's identity (10). Accordingly, non-native CRP has also been found to be protective against pneumococcal infection (117–119). Although it is not clear what structure on immobilized proteins is recognized by non-native CRP, it has been proposed that an amyloid-like structure is formed on all such proteins and that is what is being recognized by non-native CRP, consistent with the hypothesis that CRP is a pattern recognition molecule of the innate immune system (10). Indeed, an amyloid-like structure appears on LDL by oxidation (120, 121). Non-native CRP may serve as a tool to investigate the functions of CRP in every inflammatory disease involving deposition and aggregation of proteins, such as amyloidosis and autoimmune diseases (122). CRP may have been conserved throughout evolution for protection against disease and toxicity caused by protein misfolding and conformationally altered pathogenic proteins (123, 124).

Considering all the properties of all forms of CRP, it can be said that CRP possesses the functionality of a host defense molecule against not only atherosclerosis but against all diseases caused by proteins when proteins behave like a pathogen or a toxic molecule, in a life cycle that begins as free CRP in circulation and ends in ligand-bound mCRP at sites of inflammation via an intermediate stage of non-native pentamers. If it is validated through further experimentation employing mice transgenic for non-native CRP that non-native CRP is beneficial, the focus should be on the designing and synthesis of a small-molecule compound to target CRP with the goal of changing the conformation of endogenous CRP, which would be preferred over using recombinant non-native CRP as a biologic to treat diseases caused by pathogenic proteins such as ox-LDL.

## Author Contributions

All authors listed have made a substantial, direct and intellectual contribution to the work, and approved it for publication.

### Conflict of Interest Statement

The authors declare that the research was conducted in the absence of any commercial or financial relationships that could be construed as a potential conflict of interest.

## Abbreviations

CRP: C-reactive protein
CRP or native CRP: native pentameric CRP
non-native CRP: non-native pentameric CRP
mCRP: monomeric CRP
LDL: low-density lipoprotein
ox-LDL: oxidized LDL
E-LDL: enzymatically-modified LDL
PCh: phosphocholine.
